# ﻿Pollen transfer efficiency in *Erica* depends on type of pollinator

**DOI:** 10.3897/phytokeys.244.107288

**Published:** 2024-07-23

**Authors:** Sam McCarren, Jeremy J. Midgley, Anina Coetzee, Steven D. Johnson

**Affiliations:** 1 Department of Biological Sciences, University of Cape Town, 7701 Cape Town, South Africa University of Cape Town Cape Town South Africa; 2 Department of Conservation Management, Nelson Mandela University, 6530 George, South Africa Nelson Mandela University George South Africa; 3 Centre for Functional Biodiversity School of Life Sciences, University of Kwazulu-Natal, 3209 Pietermaritzburg, South Africa University of Kwazulu-Natal Pietermaritzburg South Africa

**Keywords:** bee, bird, exserted anthers, long-proboscid fly

## Abstract

Pollen transfer efficiency (PTE; the proportion of pollen removed from flowers that reaches conspecific stigmas) is expected to vary with the type of pollinator and flower morphology, and to influence male siring success. Many species in the genus *Erica* are pollinated by bees (which consume pollen and should thus lower PTE) but during its radiation in the Cape, several independent shifts to both sunbird and long-proboscid fly (LP fly) pollinators, which do not consume pollen have taken place. Improvements in PTE could be one of the factors driving these pollinator shifts. PTE data for 15 *Erica* species (five for each of the three pollinator types) were collected and compared in relation to type of pollinator and anther exsertion. LP fly- and bird-pollinated species had higher PTE in comparison with bee-pollinated species. Species with inserted anthers had higher PTE than those with exserted anthers. This suggests that sunbirds and LP flies are more efficient pollinators than bees. Additionally, the study suggests that insertion of anthers within the corolla tube can reduce pollen losses.

## ﻿Introduction

The reproductive success and number of seeds produced in flowering plants strongly depends on the efficiency of pollen removal and its subsequent deposition on conspecific stigmas (Johnson and Harder 2023). This can be quantified through the index of pollen transfer efficiency (PTE), which reflects the proportion of pollen removed from flowers that reaches stigmas (Johnson et al. 2005), and is expected to vary with type of pollinator (Laverty and Plowright 1988; Shuttleworth and Johnson 2008; Willmer et al. 2017). For example, it was shown that PTE was higher in a hummingbird-adapted *Penstemon* than in a bee-adapted congener (Castellanos et al. 2003). While it has been suggested that bees often act as pollen thieves by collecting pollen without effectively pollinating flowers (Hargreaves et al. 2009), e.g., to consume it or due to their grooming behaviour which cleans pollen off them, there have not been direct comparisons of the pollen transfer efficiencies of bees, nectarivorous birds and long-proboscid flies (LP flies), which are all important pollinator groups in southern Africa (Goldblatt and Manning 2000). In general, increased PTE could explain why shifts to non-grooming pollinators such as birds and LP flies have occurred, even though these shifts require investment in larger flowers (Castellanos et al. 2003).

The genus *Erica* is highly suitable for studying differences in PTE between pollinator groups because of its species diversity (ca. 700 in South Africa) and diversity of pollinators (van der Niet 2021). Most *Erica* species are pollinated by short-tongued insects, such as bees, but during its radiation in the Cape of South Africa, several independent shifts to both sunbird and LP fly pollination syndrome have taken place in the genus (Pirie et al. 2011). Nevertheless, it is not understood what factors precipitated these shifts and, if there are any differences in PTE, then those could be one of the selective factors driving the morphological changes associated with pollinator shifts (Kobayashi et al. 1997).

*Erica* species pollinated by bees or other short-tongued insects are the largest group in the genus (Rebelo et al. 1985). They typically produce many small flowers with low volumes of nectar (Bouman et al. 2017). Bees as pollinators tend to effect lower pollen carryover among plants compared to other pollinators, which is most likely due to their pollen grooming behaviour (Castellanos et al. 2003; Holmquist et al. 2012). Since grooming pollen lowers the fraction of removed pollen that can land on conspecific stigmas, bee-pollinated species are likely to have lower PTE than *Erica* species with other pollinators.

Adaptations to non-bee pollinators such as sunbirds and LP flies in the genus *Erica* might incur greater flower production costs but could also increase pollination success as a trade-off. For sunbird-pollinated *Erica* species, their long corollas in a variety of colours (Rebelo and Siegfried 1985), a higher volume of nectar (Rebelo et al. 1984) and the provision of a perch (Siegfried et al. 1985) might be costly. Similarly, LP fly-pollinated *Erica* species also tend to have long sticky corollas (McCarren et al. 2021a) and produce nectar high in volume and concentration (Goldblatt and Manning 2000; McCarren et al. 2023). Further, they reflect light in the ultraviolet range (McCarren et al. 2021b) which might make them more vulnerable to damage by UV-B radiation due to the lack of protection by ultraviolet-absorbing compounds (Llorens et al. 2015). Additionally, LP flies visit *Erica* flowers infrequently, resulting in relatively low pollination rates (McCarren et al. 2023). The adaptations mentioned above are likely to make both bird and LP fly pollination more energetically expensive for the plants and thus it is expected that these pollinators must confer other fitness benefits to compensate for the associated costs (Stiles 1978). These benefits could include the pollinators moving greater distances between plants while foraging, higher pollen carryover, limited pollen grooming (Krauss et al. 2017) and increased pollination accuracy (Armbruster et al. 2009). Therefore, *Erica* species pollinated by non-bees are expected to have relatively high PTE.

Many *Erica* species have exserted anthers, which appears to be a trait that evolved independently in multiple lineages (Pirie et al. 2011). Having exserted anthers can cause more pollen to be removed during the first pollinator visit (Harder and Barrett 1993), which could be beneficial when pollinator visits are rare or unpredictable. The function of exserted anthers in bird-pollinated species is likely to place pollen on their head feathers once the bill is fully inserted in the tube (Ojeda et al. 2016). Because pollen is less likely to be lost during transport on feathers than on the smooth bill of birds, *Erica* species with exserted anthers are expected to have higher PTE compared to species with included anthers. However, exserted anthers are also found in some bee-pollinated species and this may be associated with pollen being offered as a reward, which may decrease PTE. Therefore, it is unclear what the effect of anther exsertion is on PTE overall.

The aims of this study were to (a) compare PTE between bee-, bird- and LP fly-pollinated *Erica* species, and (b) compare PTE between *Erica* species with exserted and included anthers. This was addressed by collecting PTE data for 15 *Erica* species in total, with five species per type of pollinator, six species with exserted anthers and nine with included anthers.

## ﻿Methods

### ﻿Sample collection and analysis

A total of 15 *Erica* species were sampled in the Cape Floristic Region of South Africa with five species for each of three pollination syndromes: bird, LP fly and bee (Table 1). Syndrome classification was based on flower morphology (Rebelo et al. 1985) and confirmed by literature (Rebelo et al. 1984; Lombardi 2014; van der Niet et al. 2014; Bouman et al. 2017; Lombardi et al. 2021; Pauw 2022; McCarren et al. 2023), iNaturalist records and pollinator observations. Six of these species have exserted anthers, with three of them bee-pollinated and three bird-pollinated. Per species, 30 flowers were sampled, including ten unvisited flowers, which can be recognised by their intact anther ring (Geerts and Pauw 2011) and 20 flowers in late anthesis from different plants, whose corollas had begun to wilt (and therefore had no further opportunity to be pollinated). Flowers were randomly collected from different individuals. The anthers from undisturbed flowers, and the anthers and stigma from flowers in late anthesis were separated and kept individually in Eppendorf tubes. In the laboratory, the anthers were suspended in 1 ml ethanol and stained with fuchsin. The pollen suspension was homogenised with a vortex and then immediately four 20 μl drops from the sample were placed on a slide to count the pollen grains under a Leica DM500 compound microscope at 100× magnification.

**Table 1.** Mean number of pollen grains deposited and removed ± standard deviation in 15 *Erica* species, the calculated PTE, their type of pollinator (long-proboscid fly = LP fly), anther exsertion, sample location and time.

Species	Pollen deposition	Pollen removal	Pollen production	PTE (%)	pollinator	Anther exsertion	Sample location	Month
*E.aristataaristata* Andrews	199 ± 136	45130 ± 12752	46495 ± 15151	0.4	LP fly (Rebelo et al. 1985; Lombardi et al. 2021)	included	Vogelgat	September
*E.cristata* Dulfer	91 ± 56	3541 ± 1681	3748 ± 1969	2.6	LP fly (Rebelo et al. 1985, iNaturalist record 39626162)	included	Vogelgat	March
*E.retorta* Montin	362 ± 236	18873 ± 17444	19125 ± 17740	1.9	LP fly (Rebelo et al. 1985)	included	Kogelberg	November
*E.ampullaceaampullacea* Curtis	830 ± 296	38800 ± 29292	46020 ± 35044	2.1	LP fly (Rebelo et al. 1985; McCarren et al. 2023, observations)	included	Boskloof	August
*E.fastigiatacoventryi* Bolus	222 ± 149	2969 ± 2084	3461 ± 2483	7.5	LP fly (Rebelo et al. 1985; Pauw 2022, iNaturalist record 11115439)	included	Vogelgat	September
*E.sessiliflora* L.f.	224 ± 208	15549 ± 9200	16060 ± 9845	1.4	bird (Rebelo et al. 1985; Lombardi 2014, observations)	included	Vogelgat	September
*E.viscariapustulata* L.	790 ± 272	14205 ± 8768	14400 ± 8939	5.6	bird (observations)	included	Vogelgat	March
*E.plukenetiiplukenetii* L.	206 ± 72	35935 ± 12152	37695 ± 14016	0.6	bird (Rebelo et al. 1984, 1985, van der Niet et al. 2014, observations)	exserted	Vogelgat	September
*E.monadelpha* Andrews	246 ± 188	14003 ± 8972	15185 ± 10414	1.8	bird (Rebelo et al. 1985, observations)	exserted	Fernkloof	June
*E.melastomamelastoma* Andrews	548 ± 248	36023 ± 15724	39405 ± 23879	1.5	bird (observations)	exserted	Vogelgat	September
*E.imbricata* L.	49 ± 36	5480 ± 3132	5635 ± 3497	0.9	bee (Rebelo et al. 1985; Bouman et al. 2017, observations)	exserted	Vogelgat	June
*E.laeta* Bartl.	168 ± 120	3488 ± 1596	3550 ± 1766	4.8	bee (Rebelo et al. 1985, observations)	included	Vogelgat	March
*E.labialis* Salisb.	8 ± 4	7453 ± 1873	7465 ± 1899	0.1	bee (Bouman et al. 2017, observations)	exserted	Vogelgat	March
*E.ericoides* L.	44 ± 21	9880 ± 3008	5130 ± 5130	0.4	bee (observations)	exserted	Table Mountain National Park	December
*E.quadrangularis* Salisb.	198 ± 108	4785 ± 4868	9928 ± 3068	4.1	bee (Rebelo et al. 1985, observations)	included	Hottentot Hollands	December

The stigmas were mounted in molten fuchsin gel on a microscope slide using a cover slip. Pollen was counted under a Leica DM500 compound microscope at 100× magnification. There was no noticeable altitudinal or spatial clustering of species sharing types of pollinators, and at most of the sites no other *Erica* species from the same pollination syndrome were in flower at the time, except for some bee-pollinated species which co-flowered with one other bee-pollinated *Erica*. However, even when sharing pollinators, the high levels of flower constancy exhibited by bees cause high pollen purity (i.e., pollen from only one species) on the stigmas of co-occurring *Erica* species (van der Niet et al. 2020), and the difference in pollen aggregation for the co-flowering species (monads and tetrads) would have indicated heterospecific pollen transfer. Thus, it was assumed that the pollen counted on the stigmas was monospecific.

### ﻿Statistical analysis

Since most *Erica* species produce pollen in tetrads (Wrońska-Pilarek et al. 2018), the number of pollen tetrads in the anthers and on the stigmas was further multiplied by four to calculate the total number of pollen grains, except for *E.cristata*, *E.ericoides*, *E.fastigiata* and *E.labialis* since those species produce pollen monads. Pollen removal was calculated as mean pollen removal per species by subtracting the mean pollen remaining in all disturbed anthers from the mean pollen produced in all unvisited anthers. Pollen transfer efficiency (PTE) was calculated for each species following the formula PTE= mean pollen deposition/mean pollen removal (Johnson et al. 2005). Statistical analyses were carried out in R (R Core Team 2022) by fitting generalised linear models with negative binomial error structure and using the log link function from the package ‘MASS’ (Ripley et al. 2019). Due to the many problems with analysing ratios (Johnson and Harder 2023), the variation in PTE was not tested directly. Instead pollen deposition (the response variable) was explored in relation to type of pollinator as explanatory variable with pollen removal as a covariate. Pollen removal was log transformed prior to the analysis so that it had the same scale of measurement as the response variable. The same model was repeated with anther exsertion as the explanatory variable and both pollen removal and type of pollinator as additional predictors. Since no LP fly-pollinated flowers had exserted anthers, those species were excluded from the analysis testing for an effect of anther exsertion. Due to the small sample size and consequently low statistical power, the interaction of type of pollinator and anther exsertion was not included in the model. Additionally, pollen production in relation to PTE, as well as pollen production and deposition in relation to type of pollinator, were modelled. The proportion of pollen removed was also modelled in response to type of pollinator using a beta GLM from the package ‘betareg’ (Cribari-Neto and Zeileis 2010). A beta distribution was used here since the model had a proportion as its response variable. The models comparing pollen production, pollen deposition and proportion of pollen deposited in relation to type of pollinator were repeated for bird- and bee-pollinated species only with anther exsertion as an additional predictor. Tukey’s post hoc tests from the package ‘emmeans’ (Lenth and Lenth 2018) were used to identify the differences for models with significant terms.

## ﻿Results

**Figure 1.**
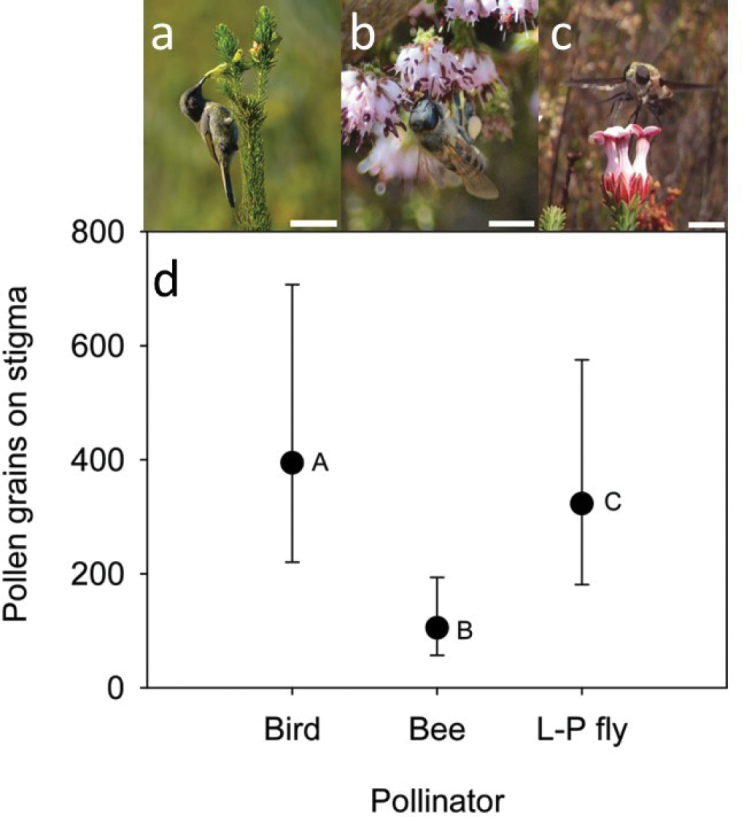
**a** Orange-breasted sunbird (*Anthobaphesviolacea*) visiting the bird-pollinated *Ericaviscaria***b** honeybee (*Apismellifera*) visiting the bee-pollinated *Ericaericoides***c** long-proboscid fly (*Prosoecawestermanni*) visiting the LP fly-pollinated *Ericaampullacea***d** mean (±95% confidence interval) pollen deposition for *Erica* species in relation to their type of pollinator after adjusting for pollen removal. Means that share letters are not significantly different. Scale bars: 40 mm (**a**); 5 mm (**b**); 15 mm (**c**).

**Figure 2.**
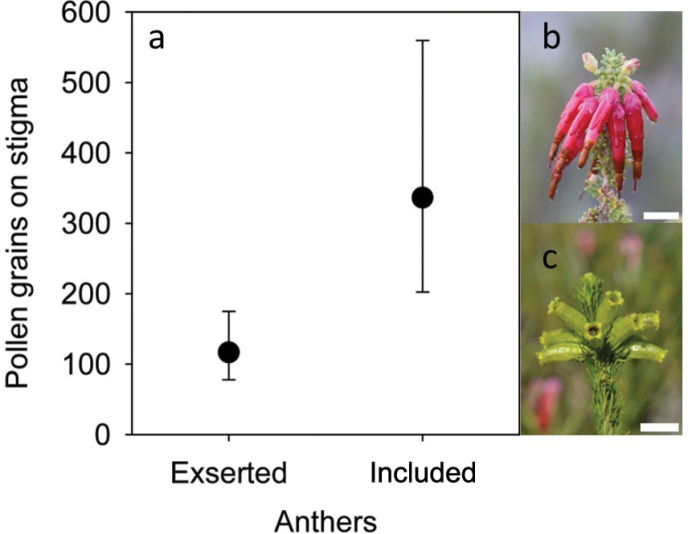
**a** Mean (±95% confidence interval) pollen deposition for *Erica* species in relation to their anther exsertion after adjusting for pollen removal and type of pollinator **b** exserted anthers in *E.monadelpha***c** included anthers in *E.viscaria*. Scale bars: 10 mm (**b**); 15 mm (**c**).

Almost all sampled flowers (98.3%) had at least some pollen deposited on their stigma and 85% had some pollen remaining in their anthers in late anthesis, so that on average 5.1% of the total pollen produced remained in the anthers. The recorded PTE values (Table 1) ranged from 0.1% to 7.5%. There was a significant effect of type of pollinator on pollen deposition after adjusting for pollen removal (ꭓ^2^ = 6.64, df = 2, p= 0.036, Fig. 1). Pollen deposition (adjusted for pollen removal) was about four-fold greater in bird- and LP fly-pollinated species than it was in bee-pollinated species (Fig. 1). The partial regression coefficient associated with removal did not differ significantly from zero (b= 0.070, Z= 0.346, p= 0.729), indicating that pollen deposition did not vary with removal. The post-hoc test showed that mean adjusted pollen deposition in bee-pollinated species was significantly less than that for both bird- (Z= 2.86, p= 0.012) and LP fly-pollinated species (Z= 2.69, p= 0.020), while there was no difference in pollen deposition between bird- and LP fly-pollinated species (Z= 0.40, p= 0.917). In the model with pollen deposition in response to anther exsertion, adjusted for both pollen removal and type of pollinator, pollen deposition was lower for species with exserted anthers than for species with included anthers (ꭓ^2^ = 5.04, df= 1, p= 0.025, Fig. 2). In this model, the partial regression coefficient associated with removal also did not differ significantly from zero (b= - 0.140, Z= 0.456, p= 0.648) further supporting that pollen deposition did not vary with removal. Pollen deposition in response to anther exsertion still differed between bird- and bee-pollinated species after accounting for the differences in anther position (ꭓ^2^ = 13.18, df= 1, p< 0.001). There was a negative relationship between pollen production and PTE (ꭓ^2^ = 5.57, df= 2, p= 0.018), i.e. PTE was lower for species producing large quantities of pollen and higher for species producing fewer grains. Pollen production (ꭓ^2^ = 11.30, df= 2, p= 0.004) and deposition differed (ꭓ^2^ = 9.55, df= 2, p= 0.008) significantly between types of pollinators. This was due to both bird- and LP fly-pollinated species producing (bird-pollinated: Z= 3.25, p= 0.003; LP fly-pollinated: Z= 3.17, p= 0.004) and receiving (bird-pollinated: Z= 3.11, p= 0.005; LP fly-pollinated: Z= 1.30, p= 0.016) more pollen than bee-pollinated species. The proportion of pollen removed did not vary among types of pollinators (ꭓ^2^ = 4.61, df= 2, p= 0.099). Pollen production was higher in species with exserted anthers (ꭓ^2^ = 12.31, df= 1, p< 0.001) and in this model bird-pollinated species had higher pollen production than bee-pollinated species (ꭓ^2^ = 55.81, df= 1, p< 0.001). Pollen deposition, on the other hand, was lower in species with exserted anthers (ꭓ^2^ = 7.65, df= 1, p= 0.006) while bird-pollinated species still received more pollen than bee-pollinated species (ꭓ^2^ = 19.16, df= 1, p< 0.001). The proportion of pollen removed did not differ between different anther positions (ꭓ^2^ = 0.50, df= 1, p< 0.482) but it remained higher for bee-pollinated species compared to bird-pollinated species, as in the model above (ꭓ^2^ = 5.34, df= 1, p= 0.021).

## ﻿Discussion

PTE in the sampled *Erica* species averaged 2.4%, which is mostly higher than in other plants with granular pollen, for which PTE is typically <1% (Harder and Johnson 2008). This might be related to the relatively specialized pollination systems of the sampled *Erica* species. However, even though relatively high for plants with granular pollen, PTE in the sampled *Erica* species is still relatively low compared to values of up to 40% recorded for some orchids (Johnson et al. 2005; Hobbhahn and Harder 2016) and asclepiads (Shuttleworth and Johnson 2008) that produce aggregated pollen in the form of pollinia. There is generally a negative relationship between PTE and pollen production (Gong and Huang 2014; Harder and Johnson 2023), which suggests that production of pollen may evolve in relation to the risk of it being lost in transit between flowers (Harder and Johnson 2023). Relatively low pollen-ovule ratios in *Erica* may reflect the aggregation of pollen in tetrads and high PTE in this genus (Harder and Johnson 2008; Arendse et al. 2021). However, the expected association between pollen-ovule ratios and type of pollinator has not been confirmed in *Erica* (Arendse et al. 2021)

As expected, we found relatively low PTE in bee-pollinated *Erica* species and higher PTE in both bird- and LP fly-pollinated species. This supports the idea that nectarivorous birds are more efficient pollinators than bees (Castellanos et al. 2003). This study is one of the first to compare PTE between LP flies and other pollinators (see also Johnson and Harder 2023), and our observation that PTE of LP fly-pollinated species is higher than in bee-pollinated species, but does not differ from bird-pollinated species, is consistent with the idea that non-grooming pollinators confer greater PTE to the plants that they pollinate (Johnson and Harder 2023). However, the distinguishing feature of *Erica* species pollinated by LP flies could be that their anthers are always included, rather than the characteristics of their pollinator. Since the type of pollinator and anther exsertion are confounded for LP fly-pollinated species, experiments that specifically tease apart these factors are necessary to make unequivocal statements.

Seed production of *Erica* species pollinated by LP flies is often pollen-limited (McCarren et al. 2023). This is seemingly in contradiction to the results of this study which showed that they receive more pollen than bee-pollinated species and have high PTE with most stigmas appearing to be saturated with pollen grains. It is possible that geitonogamous pollen transfer, as a result of LP flies visiting several flowers per plant, could play a role in clogging stigmas with self-pollen reducing the number of seeds produced (Coetzee et al. 2020), and this effect would be exacerbated in the case of LP fly-pollinated *Erica* species that have late-acting self-incompatibility as commonly found in the genus (Arendse et al. 2021). While PTE methodology cannot discriminate between cross- and self-pollen, the risk of geitonogamous selfing is a general disadvantage of producing many flowers per plant (de Jong et al. 1992). However, because LP fly- and bird-pollinated *Erica* species tend to have fewer flowers per plant than those pollinated by bees, it seems unlikely that their higher levels of PTE would be caused by geitonogamous pollen transfer. It is more likely that the link between PTE and seed production is weak, since PTE is a measure of male fitness, while seed production is a measure of female fitness and might be impacted by additional traits, such as differences in style length and number of ovules.

This study shows that in most cases pollen still can be found in *Erica* anthers in late anthesis. The first visit to a flower causes the anther ring to break and release an explosive puff of pollen (Geerts and Pauw 2011), which might cause a large amount of pollen to be removed, but successive visits could still place some pollen on the pollinator. It has been predicted that increased pollen removal by one pollinator causes diminishing returns in pollen deposition (Harder and Thomson 1989; Harder and Wilson 1994) which would likely make it inefficient to place all or most pollen on the first visitor unless there are very few pollinator visits. Thus, in *Erica* the exploding anther ring might be an advantage when visitation rates are low like it has been reported e.g. for LP fly-pollinated species (McCarren et al. 2023), or it could increase pollen placement in hard-to-reach sites on the pollinator bodies where it is less likely to be groomed off.

We found that *Erica* species with exserted anthers have lower PTE than species with included anthers. Pollen removal typically increases with anther exsertion (Conner et al. 1995), but we found no difference in the proportion of pollen removed in relation to anther exsertion. *Erica* species with exserted anthers do, however, produce higher amounts of pollen but this increase in production does not coincide with an increase in deposition, which indicates that more of the removed pollen is lost to the environment. It is not clear how the pollen is lost, but once the anther ring has been broken, it could more easily be blown away by wind and washed away by rain, while in species with included anthers the pollen would likely remain inside the floral tube where it is still available to pollinators. Further, it might be easier for bees and other pollen thieves to collect and rob pollen from exserted anthers. Having exserted anthers thus imposes a cost since the plants produce more pollen while less of it ends up on conspecific stigmas. This could be a trade-off against other benefits like a different pollen placement site, which can reduce the risk of the stigma receiving heterospecific pollen (Manning and Goldblatt 1997; Muchhala and Thomson 2012).

With increasing pollen production, PTE decreases for *Erica* species, which is consistent with findings from other studies (Harder and Johnson 2023). This could be caused by plant species with less efficient pollinators compensating for low PTE with increased pollen production as a strategy that ensures reproductive success.

This study has shown that PTE differs among *Erica* species with different types of pollinators, as well as in relation to anther exsertion. These differences in PTE are likely the result of costs and benefits associated with different reproductive strategies, which in turn might have driven pollinator shifts and consequently speciation in the genus *Erica*.

## ﻿Additional information

### Author ORCIDs

Sam McCarren https://orcid.org/0000-0002-3861-0811

Jeremy J. Midgley https://orcid.org/0000-0001-7831-2301

Anina Coetzee https://orcid.org/0000-0002-1646-557X

Steven D. Johnson https://orcid.org/0000-0002-5114-5862

### Data availability

All of the data that support the findings of this study are available in the main text.
